# Establishment of an easy and straight forward heparinase protocol to analyse circulating and myocardial tissue micro-RNA during coronary artery-bypass-graft surgery

**DOI:** 10.1038/s41598-018-19748-6

**Published:** 2018-01-22

**Authors:** Andrea Engler, Florian Dreja, Sarah Köberle, Matthias Thielmann, Jürgen Peters, Ulrich H. Frey

**Affiliations:** 1 Klinik für Anästhesiologie und Intensivmedizin, Universität Duisburg-Essen and Universitätsklinikum Essen, Essen, 45147 Germany; 2 Klinik für Thorax- und kardiovaskuläre Chirurgie, Universität Duisburg-Essen and Universitätsklinikum Essen, Essen, 45147 Germany

## Abstract

Coronary artery-bypass-graft (CABG) surgery is associated with myocardial damage and increased blood concentrations of circulating microRNAs (miRNA). However, whether and to what extent these miRNAs relate to cardiac tissue miRNA expression have not yet been explored. Since plasma miRNA quantification in samples from cardiopulmonary bypass (CPB) patients is severely hampered by heparin, we established and validated successfully a protocol to reliably measure miRNA in 49 heparinized patients undergoing CABG so as to investigate the relationship between circulating and right atrial miRNAs. Plasma and right atrial expression of miR-1, miR-133a, miR-423-5p, and miR-499 were measured before and after CPB, as well as miRNAs in plasma 24 h thereafter. All plasma miRNAs increased significantly with surgery while cardiac tissue expression of only miR-133a (1.4-fold; p = 0.003) and miR-423-5p (1.3 fold; p = 0.025) increased as well. Right atrial and plasma miR-133a expression correlated positively before CPB (r = 0.288, p = 0.045) but miR-499 expression inversely (r = −0.484, p = 0.0004). There was a strong association between plasma miR-133a and miR-499 concentrations and postoperative troponin I concentrations, the marker for myocardial damage. Increased myocardial miR-133a and miR-423-5p expression together with unchanged miR-1 and miR-499 expression might suggest active release of these miRNAs rather than their origin from damaged cells.

## Introduction

MicroRNAs (miRNAs) are small (20–24 nt) non-coding RNAs that regulate mRNA expression mostly at the post-transcriptional level. Circulating miRNAs are protected against degradation by binding to RNA binding proteins like Argonaute 2^1^, nucleophosmin^2^, or HDL^3^, or they are present in extracellular vesicles like exosomes^4^ or microparticles^5^. In recent years, the importance of circulating miRNAs as potential biomarkers for various disease states has been established and extensively reviewed, e.g.^6–9^. It has been shown that increased concentrations of circulating miRNAs are associated with cardiovascular conditions like acute coronary syndrome (ACS)^10^, acute myocardial infarction (AMI)^11,12^, or heart failure (HF)^13^. However, little is known about their release and transport mechanisms.

Coronary artery-bypass-graft (CABG) surgery is intrinsically associated with myocardial damage and miRNAs that have been associated with ACS, AMI or HF are altered as well during surgery^14–16^. In a mouse model of myocardial infarction, expression of miR-1, miR-133a, miR-208, and miR-499 is decreased in infarcted myocardium and it has been suggested that increased concentrations of serum miR-133a in patients derive from injured myocardium^17^. In addition, miR-1 and miR-133a expression is decreased in autopsy samples of infarcted heart tissue^18^. We recently showed, that cardiac miR-133a expression in patients undergoing CABG surgery decreased as severity of HF increased^19^. Moreover, a miRNA array study revealed miR-423-5p as a predictor for HF^20^ and miR-423-5p is enriched in the pericardial fluid of CABG patients^15^.

While it is, therefore, compelling to investigate the relation of plasma and cardiac tissue miRNA expression to shed light on the potential origin of these miRNA, high heparin dosages used during cardiopulmonary bypass (CPB) for CABG and other cardiac surgeries inhibit reverse transcription reactions and the *Taq* DNA polymerase^21–24^. Since heparin co-purifies with nucleic acids, it also interferes with miRNA quantification by the quantitative polymerase chain reaction (qPCR). It has been demonstrated that intravenous heparin alters plasma miRNA quantification depending on its dose and sampling time^25,26^ and an alternative normalization strategy has been proposed^26^. For mRNA quantification, the use of lithium chloride precipitation^27^ or heparinase I incubation^23,28^ to remove the inhibitorily acting heparin has been proposed.

To analyse circulating plasma miRNAs, we adapted a protocol for qPCR detection of 18S rRNA and other mRNA targets in heparinized samples^28^. Our protocol implements heparinase I treatment of RNA isolated from heparinized plasma samples using the buffer and RNase inhibitor included in the commercially available reverse transcription kit immediately prior reverse transcription so as to overcome the inhibitory effect of heparin and to allow reliable miRNA detection by qPCR.

Specifically, we explored the relation between myocardial and plasma expression of miR-1, miR-133a, miR-499, and miR-423-5p. These miRNAs are specifically expressed in cardiac and skeletal muscle, are enriched in cardiomyocytes, and have been associated with cardiovascular disease.

We hypothesized that circulating miRNAs may reflect their expression in human cardiac muscle and relate to cardiac ischemia/reperfusion injury, as assessed by troponin I concentrations, and that the comparison of tissue and plasma miRNA expression may hint to the origin of these circulating miRNAs.

## Results

**Figure 1:**
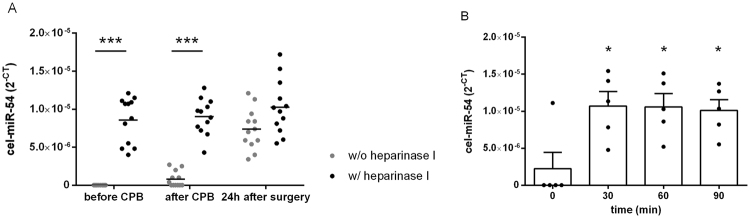
Plasma cel-miR-54 expression in samples from patients undergoing CABG. (**A**) Effect of heparinase I incubation on relative plasma cel-miR-54 concentrations (2^−CT^). Cel-miR-54 expression was determined by qPCR (n = 12). RNA samples before and after CPB, and from 24 h after surgery were either untreated or treated with 1 U heparinase I prior to reverse transcription and qPCR. Statistics: Repeated measure ANOVA followed by *post hoc* t-tests; ***P < 0.001. (**B**) Relative plasma cel-miR-54 concentrations (2^−CT^) in samples before CPB after heparinase I treatment for different time periods. Prior to reverse transcription, miRNA samples were incubated with heparinase I for the indicated time periods and then analysed for cel-miR-54 expression by qPCR (n = 5). Statistics: One-way ANOVA followed by Dunnett’s *post hoc* test; *P < 0.05 vs. 0 min.

To verify that our heparinase I treatment protocol was working properly, we first analysed plasma samples derived from twelve patients undergoing CABG surgery for the expression of the spike-in control cel-mir-54 (Fig. 1). All plasma samples were spiked with cel-mir-54 before RNA isolation. RNA samples were either left untreated or treated with 1 U heparinase I for 30 min before reverse transcription (Fig. 1A). In untreated samples the cel-mir-54 spike-in control was detectable only in six out of twelve samples from before CPB (Supplementary Table S1). In all corresponding samples after CPB cel-miR-54 was detectable, but there was great variation in the threshold cycle (C_T_) which is used for quantification ranging from 38.1 to 18.5 (mean C_T_: 25.6 ± 2.2; Supplementary Table S1). Only in the samples 24 hours after surgery, cel-mir-54 was detectable in all samples with a mean C_T_ of 17.1 ± 0.2 (Supplementary Table S1). This resulted in an apparent continuous increase in cel-miR-54 expression from samples obtained before and after CPB to those 24 hours later (Fig. 1A).

In contrast, cel-miR-54 expression in samples treated with heparinase I was detectable at similar concentrations in all samples from all sampling times (Fig. 1A), indicating efficient removal of heparin by our protocol. Mean C_T_ values for before and after CPB, and 24 h samples averaged 16.9 ± 0.2, 16.8 ± 0.1, and 16.7 ± 0.1, respectively and these values were not statistically different (Supplementary Table S1). A thirty minute heparinase I incubation was sufficient to restore the detection of the spike-in control cel-miR-54 in samples obtained before CPB to the levels of those from 24 h after surgery (Fig. 1B). Extension of the incubation time did not further enhance cel-miR-54 detection. There was no significant difference in the expression of cel-miR-54 between samples obtained after 30, 60 and 90 minutes of incubation with heparinase I and therefore, 30 minutes of incubation with heparinase I were chosen for all subsequent samples.

In addition, in plasma samples with and without heparin from healthy volunteers we could show that there was no significant difference in the expression levels of cel-miR-54 and miR-133a after our heparinase I treatment. Furthermore, in samples without heparin, the expression of cel-miR-54 and miR-133a was not influenced by our heparinase I treatment (Supplementary Figure S1).

**Figure 2:**
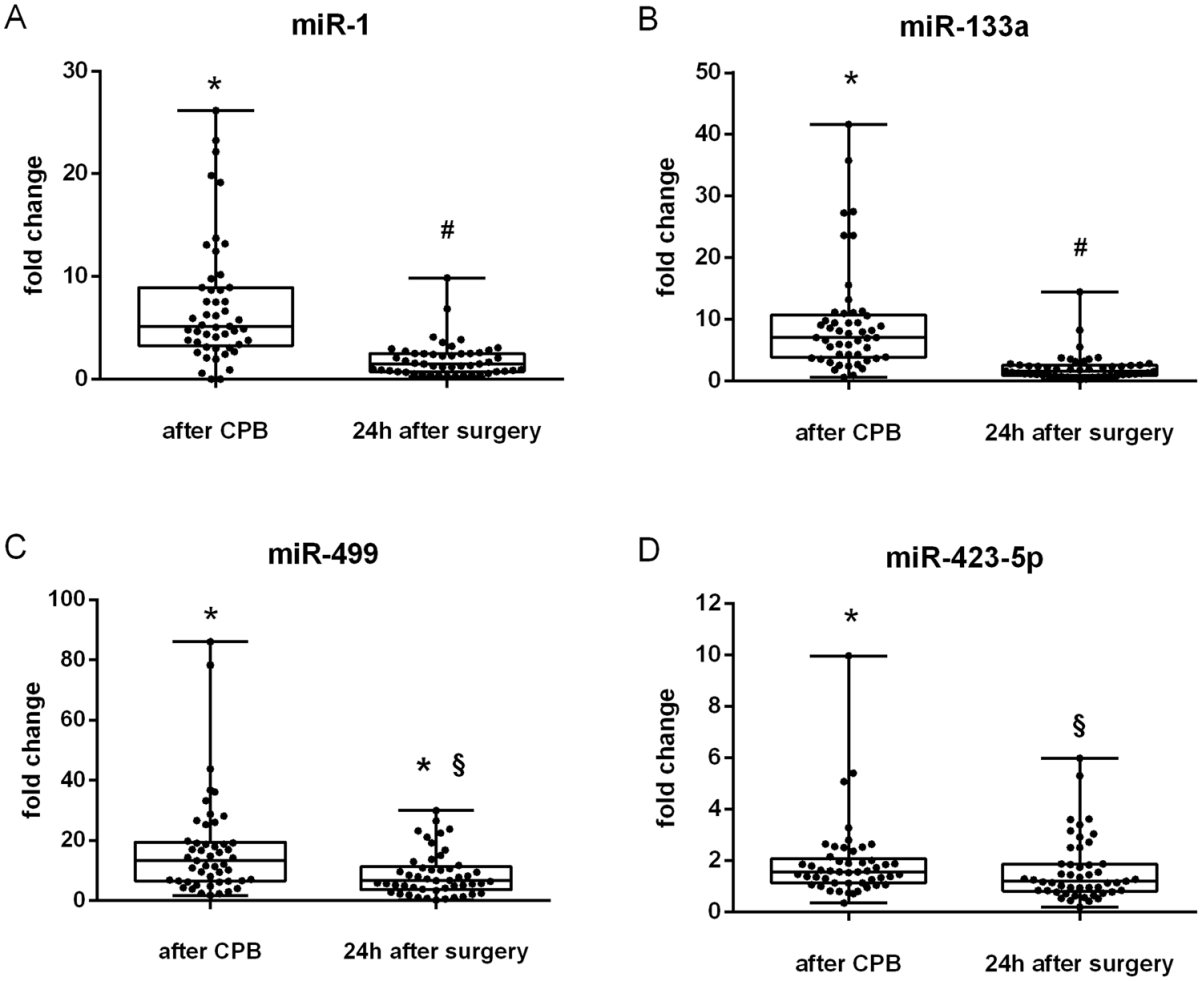
Plasma expression of miR-1, miR-133a, miR-499, and miR-423-5p after CABG. Expression fold change of plasma miR-1 (**A**), miR-133a (**B**), miR-499 (**C**), and miR-423-5p (**D**) in CABG patients (n = 49) immediately after CPB and 24 h after surgery compared to their baseline before CPB. Expression of miRNA was normalized to cel-miR-54 and is presented as fold change versus before CPB. Data are shown as box-whisker plots with median and all data points. Statistics: Friedman test followed by Dunn’s *post hoc* test; ^*^P ≤ 0.005 vs. before CPB, ^#^*P* ≤ 0.005 vs. after CPB, ^§^*P* ≤ 0.05 vs. after CPB.

Next, we analysed the expression of miR-1, miR-133a, miR-499, and miR-423-5p in all plasma samples derived during and after surgery (Fig. 2). Plasma concentrations of all four miRNAs were significantly increased immediately after CPB compared to their before CPB control, with a fold increase of miR-1, miR-133a, miR-499, and miR-423-5p of 7.3, 9.5, 17.3, and 1.9, respectively. Twenty-four hours after surgery plasma concentrations of miR-1, miR-133a, miR-499 and miR-423-5p were all significantly lower than immediately after CPB. However, while concentrations of miR-1, miR-133a, and miR-423-5p returned to their baseline concentrations before CPB, miR-499 was still significantly increased 8.8-fold compared to its baseline before CPB (Fig. 2C).

**Figure 3:**
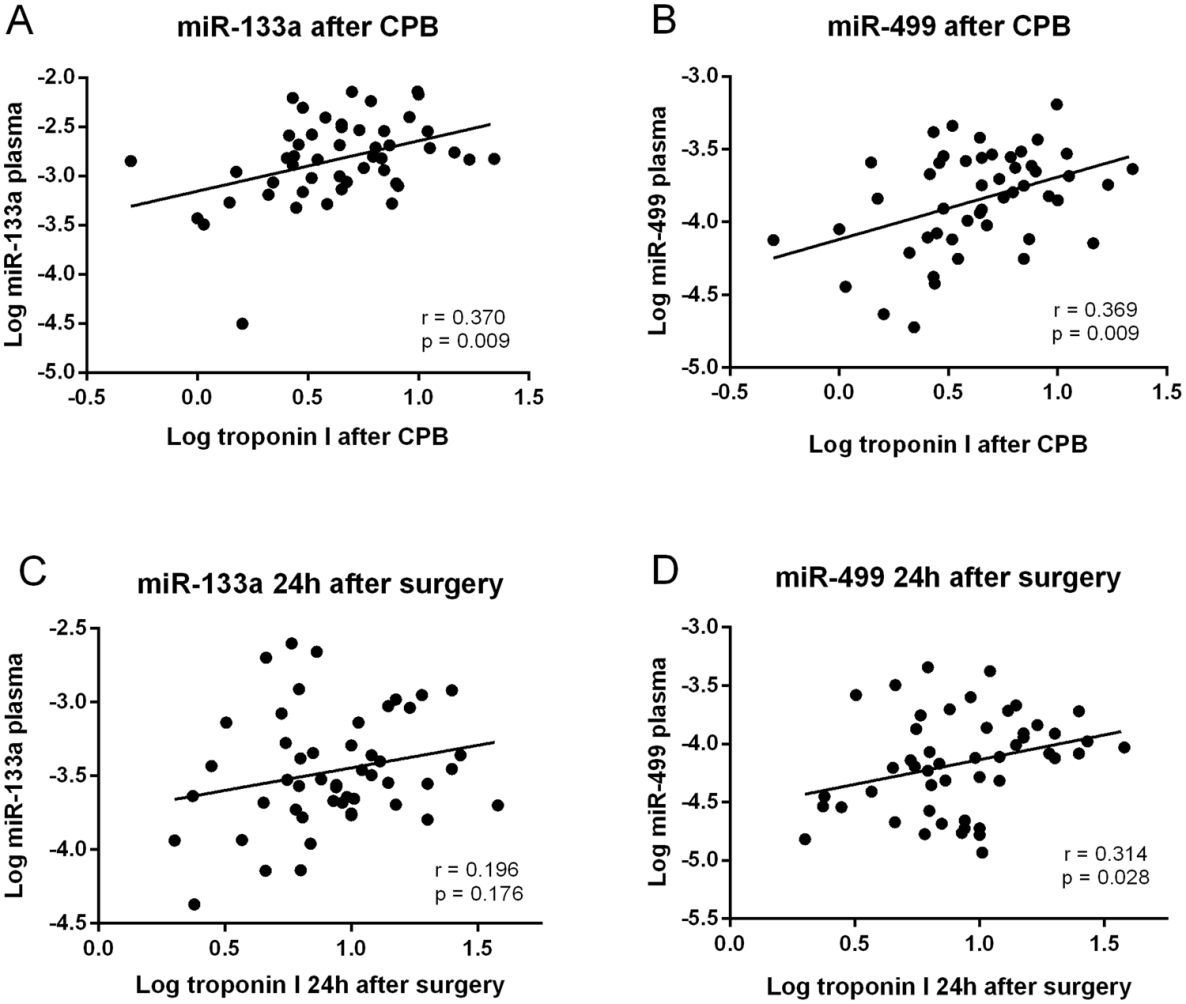
Correlations between plasma miRNA expression and troponin I concentrations. Spearman rank correlations between circulating miR-133a (**A**,**C**) or miR-499 (**B**,**D**) and troponin I concentrations immediately (**A**,**B**) and 24 h after surgery (**C**,**D**).

We then assessed the correlation between plasma expression of miR-1, miR-133a, miR-499, and miR-423-5p and serum troponin I concentrations immediately after surgery and 24 h later (Fig. 3). Troponin I concentrations peaked 24 h after surgery (Table 1). As shown in Fig. 3A,B, miR-133a and miR-499 significantly correlated with troponin I concentrations immediately after surgery. Twenty-four hours after CABG only miR-499 correlated with troponin I concentrations (Fig. 3C,D). In contrast, miR-1 and miR-423-5p expression did not correlate with troponin I concentrations at any time suggesting that only miR-133a and miR-499 relate to the extent of CABG evoked myocardial damage.

**Table 1:** Characteristics of patients.

Parameter	Patients
Age, y	66.8 ± 1.4
Male sex, n (%)	38 (77.6)
Female sex, n (%)	11 (22.4)
Physical data	
Height, cm	171.9 ± 1.3
Body weight, kg	86.2 ± 2.3
Body mass index, kg m^−2^	29.1 ± 0.7
Blood pressure, mm Hg	
Systolic	137.6 ± 3.8
Diastolic	78.6 ± 1.9
Heart rate, min^−1^	73.3 ± 2.7
NYHA class, (n = 47)	I (13), II (25), III (8), IV (1)
Clinical chemistry data	
Hemoglobin concentration, g/dL	13.8 ± 0.2
Serum creatinine concentration, mg/dL	1.3 ± 0.1
γ-GTP, IU/L	46.5 ± 7.9
Glucose concentration, mg/dL	135.7 ± 8.9
Troponin I concentration before CPB (ng/ml)	0.1 ± 0.04
Troponin I concentration upon ICU admission (ng/ml)	5.6 ± 0.6
Troponin I concentration 24 h after surgery (ng/ml)	10.4 ± 1.0
Smoking status	
Current smoker, n (%)	25 (51.0)
Former smoker, n (%)	9 (18.4)
Never smoked, n (%)	15 (30.6)
Pack year history (PY)	34.6 ± 34.7
Diabetes	
Insulin dependent, n (%)	11 (22.4)
Non-insulin dependent, n (%)	9 (18.4)
Previous myocardial infarction < 90days, n (%)	11 (22.4)
Any previous myocardial infarction, n (%)	26 (53.1)

Data are presented as means ± SEM. NYHA, New York Heart Association grade for heart failure. ICU intensive care unit.

**Figure 4:**
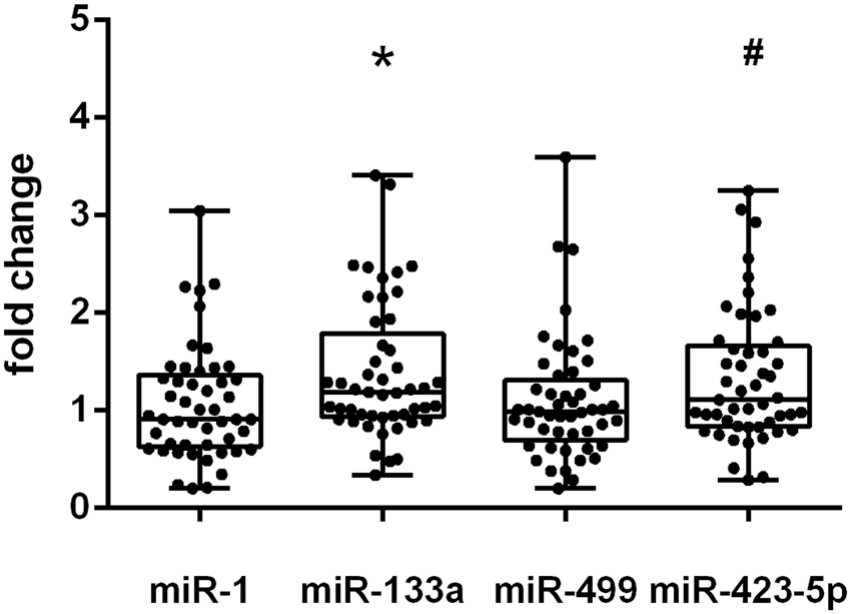
Expression of miR-133a and miR-423-5p in right atrial myocardial tissue after CABG. MiR-1, miR-133a, miR-499, and miR-423-5p changes in right atrial myocardium of CABG patients (n = 49) after CPB compared to samples before CPB. Expression of miRNA was normalized to miR-16 and is presented as fold change versus baseline before CPB. Data are shown as box-whisker plots with median and all data points. Statistics: Wilcoxon matched pairs signed rank test, *P < 0.01, ^#^*P* < 0.05 vs. before CPB.

In the next step, the expression of miR-1, miR-133a, miR-499, and miR-423-5p in right atrial (RA) myocardial samples from CABG patients was determined before and after CPB. Tissue concentrations of miR-133a and miR-423-5p after CPB significantly increased by 1.4 and 1.3-fold, respectively, when compared to baseline samples before CPB (Fig. 4). In contrast, tissue concentrations of miR-1 and miR-499 did not change.

**Figure 5:**
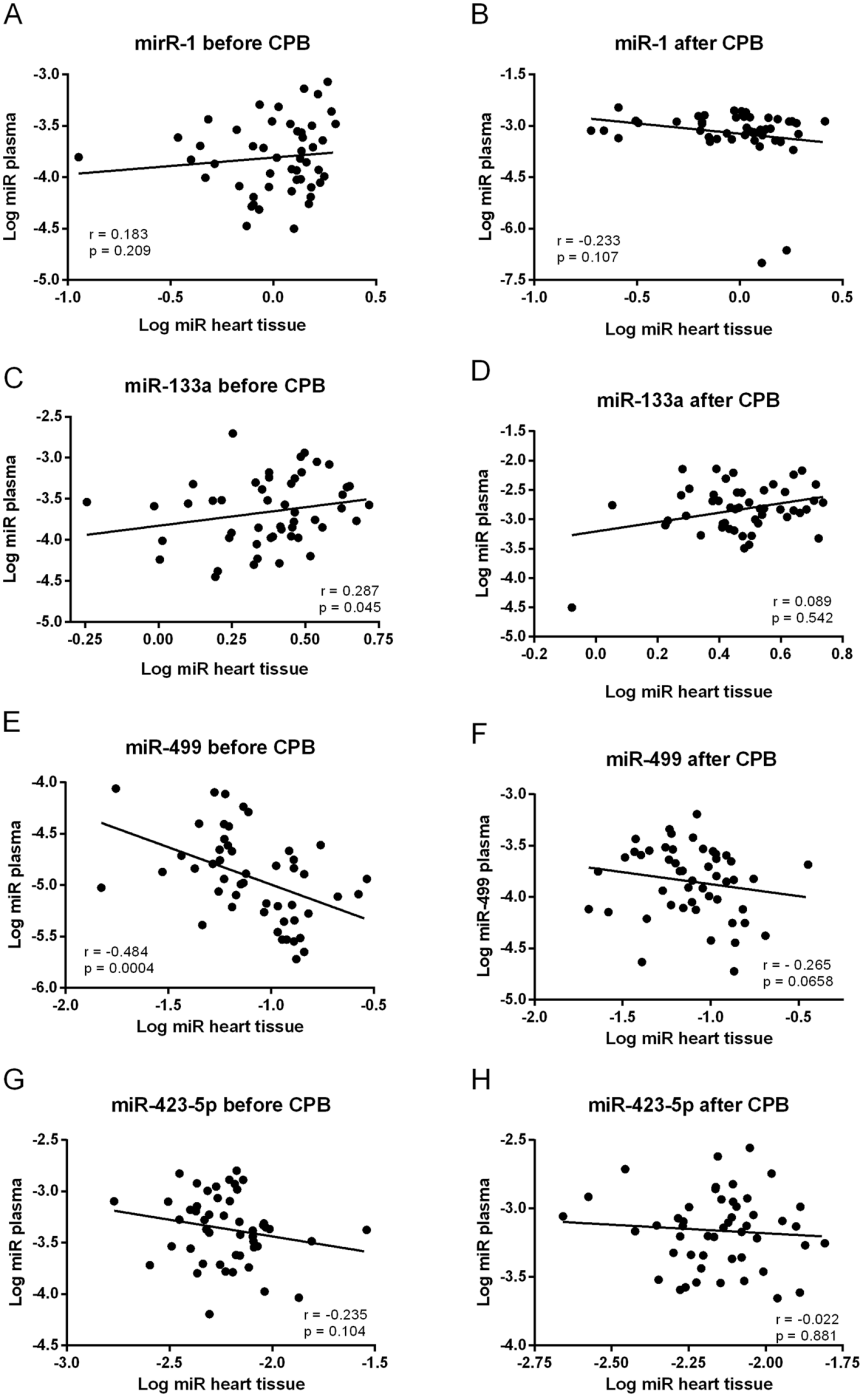
Correlations between plasma and right atrial miRNA expression before and after CABG. Spearman rank correlations between the plasma expression of miR-1 (**A**,**B**), miR-133a (**C**,**D**), miR-499 (**E**,**F**), and miR-423-5p (**G**,**H**) and their respective right atrial myocardial expression in CABG patients (n = 49) before and immediately after CPB.

We evaluated the correlation between the expression of miR-1, miR-133a, miR-499, and miR-423-5p in plasma and right atrial samples before and after CPB (Fig. 5A–H). Before CPB, plasma miR-133a and miR-499 significantly correlated with their respective tissue expression (Fig. 5C and E). However, whereas greater cardiac miR-133a expression was associated with greater miR-133a expression in plasma, an inverse correlation was seen for miR-499. Furthermore, while cardiac miR-499 expression before CBP was negatively correlated with plasma miR-499 expression after CPB, the respective miR-133a expression did not correlate (Supplementary Figure S1). No correlation was observed between plasma and cardiac tissue expression of miR-1 and miR-423-5p (Fig. 5A and G). Likewise, no correlations between plasma and heart tissue samples were observed in samples after CPB (Fig. 5B,C,F and H).

In addition, we analysed the correlation between cardiac tissue expression of miR-1, miR-133a, miR-499, and miR-423-5p both in samples before and after CPB and serum troponin I concentrations in samples after CPB and 24 h after surgery, but no significant correlations were observed (data not shown).

## Discussion

In the present study, we developed, refined, and validated a method to quantify miRNAs in heavily heparinized blood samples obtained from patients undergoing CABG surgery on cardiopulmonary bypass. Specifically, we used the *C. elegans* cel-miR-54 miRNA as a spike-in control to assess the recovery across different sample preparations and any potential inhibition caused by remaining heparin. With our heparinase I treatment protocol we were able to reliably detect cel-miR-54 at similar concentrations with very little variation throughout all sample preparations. We demonstrated that an incubation time as short as 30 minutes was sufficient to overcome any inhibitory effects of heparin. Although commercially available heparinase I is not certified as RNase-free, a prolonged incubation time up to 90 minutes did not alter the magnitude of cel-miR-54 detection and this may be attributed to the use of a RNase inhibitor in our reaction mix. In sum, our heparinase I protocol proved to remove inhibitory heparin from all patient samples and thus allows reliable miRNA quantification by qPCR in heparinized patients also during cardiac surgery.

On the clinical side, CABG surgery evoked significantly increased plasma concentrations of miR-1, miR-133a, miR-423-5p, and miR-499. This is in line with increased plasma expression of miR-1 and miR-133 after CABG reported by Emanueli *et al*.^14^. Furthermore, miR-133a and miR-499 expression in plasma after CPB showed a significant correlation with troponin I concentrations, a biomarker of myocardial cell damage and this confirms results by Yao *et al*.^29^, showing correlations of peak plasma concentrations of miR-499, miR-133a, and miR-133b with cardiac troponin I concentrations after CABG. In our patients, the correlation of plasma miR-499 and troponin I concentration persisted for at least 24 hours after surgery and a strong correlation of plasma miR-499 and troponin I concentrations even on day 4 post surgery has recently been reported^16^. Since cardiac troponins I and T are released from necrotic myocardium and both represent specific and sensitive biomarkers for myocardial damage^30^, our data thus support that miR-133a and miR-499 may also be used as circulating biomarkers for perioperative myocardial injury during CABG.

In samples obtained before cannulation for CPB and hence before aortic cross clamping and myocardial ischemia/reperfusion, miR-133a expression in plasma correlated with that in myocardial tissue. In contrast, miR-499 expression was inversely correlated, i.e., lesser myocardial miR-499 expression before CPB related to greater plasma expression. After CPB no correlation of plasma and tissue miRNAs were observed. However, lesser myocardial miR-499 expression before CPB was associated with greater plasma concentrations after CPB. Plasma miR-499 has been shown to be increased to a variable extent in acute myocardial infarction, in acute heart failure and in viral myocarditis and to reflect myocardial damage^31^. Higher plasma miR-499 expression in our patients before CPB could, therefore, be indicative of greater myocardial damage as reflected by lower tissue expression. This would be in line with our (unpublished) observation, that patients of higher NYHA functional classes tend towards lower miR-499 tissue expression before CABG.

After CPB the expression of miR-133a and miR-423-5p in heart tissue had increased by 1.4- and 1.3-fold, respectively, whereas miR-1 and miR-499 remained unchanged.

Although miR-1 and miR-133a are encoded by bicistronic pri-miRNAs, their transcription in the embryonic heart is highly regulated and controlled by the serum response factor (SRF) and myocyte enhancer factor-2 (MEF-2) transcription factors in conjunction with upstream and intragenic enhancers of miR-1/miR-133a^32,33^, integrating these miRNAs into the heart’s regulatory network^34^. In our study, the differential expression of miR-1 and miR-133a may be related to their opposing regulatory functions. While miR-1 promotes myogenic differentiation, miR-133a promotes cell growth and maintenance of an undifferentiated state^35^. Moreover, *in vitro* experiments demonstrated that miR-423-5p regulates cell proliferation and enhances cell growth in different cancers^36–38^. Therefore, one could speculate that the heart tissue’s miRNAs increased by CPB aim to counteract perioperative myocardial injury by driving cellular proliferation.

Animal studies demonstrated that myocardial miR-1, miR-133a, miR-208a, and miR-499 expression decrease after AMI in the infarcted area and its border zone and suggested that the increase in miR-1 and miR-133a expression in human serum results from miRNA release from dead cells^11,17^. In our study, however, increased miRNA plasma expression was not associated with decreased tissue miRNA concentrations, which would be the result of passive miRNA release by cell death. In addition, troponin I concentrations, a cell damage marker, was still increasing at the end of surgery and peaked only 24 h after surgery. Therefore, increased tissue miR-133a and miR-423-5p, and also unchanged tissue miR-1 and miR-499 expression, may rather suggest an active release mechanism, presumably, at least in part, via exosomes.

One potential limitation of this study is that we did not discriminate between whole plasma and exosome miRNA concentrations, which might provide information on the transport mechanisms of the miRNAs released during CABG. Emanueli *et al*., however, recently found miR-1 and miR-133 to be released both *via* exosomes and exosome-independent mechanisms in almost similar proportions after CABG, whereas miR-24 and miR-210 were predominantly released *via* exosomes^14^. Collectively, this emphasizes the need for differential approaches. Furthermore, transportation *via* extracellular vesicles other than exosomes, like microparticles or apoptotic bodies, has not been investigated in this context so far. Thus, further experiments will be necessary to elucidate the transport modes of different miRNAs.

The inhibitory effects of heparin on reverse transcription and the polymerase chain reaction have been attributed to direct interaction of heparin and DNA polymerases^39^ and/or DNA *via* divalent cations^24^ and different methodologies have been explored to address these inhibitory effects of heparin in human blood samples^23,27,40^. Kondratov *et al*. recently reported the use of calibration curves to determine amplification efficiencies and the presence of inhibitors in heparinase I treated miRNA samples from CABG patients and successful quantification of miR-1-3p and miR-208a by using a heparinase I treatment protocol for heparinized plasma quite similar to ours^41^. However, in our study, we developed a simple protocol with spike-in *C. elegans* cel-miR-54 as control, in which RNA samples treated with heparinase I could be directly used in reverse transcription. Thus, the qPCR workflow was extended by only one single step with little extra hands-on time.

In conclusion, we established and validated an easy-to-use heparinase I treatment protocol that enables reliable miRNA quantification in patient samples contaminated with high heparin concentrations. Furthermore, spiking of plasma samples with synthetic *C. elegans* cel-miR-54 allowed not only assessment of any inhibition during the qPCR workflow, but also normalization of miRNA expression and thus confirms the advantage and importance of using exogenous miRNAs for such purposes.

Finally, whereas CABG under CPB was associated with increased concentrations of circulating miR-1, miR-133a, miR-499, and miR-423-5p as well as increased miR-133a and miR-423-5p cardiac tissue expression, only circulating miR-133a and miR-499 are indicative of myocardial damage. Due to unchanged or increased cardiac tissue miRNA expression with concomitant increases in circulating miRNA concentrations, an active release mechanism for these miRNAs during cardiac surgery or a contribution of extracardiac sources is possible.

## Materials and Methods

### Patients and blood sampling

Forty-nine patients undergoing elective coronary artery bypass grafting utilizing CPB and hypothermic cardioplegic arrest under sufentanil-isoflurane anesthesia were enrolled in this prospective observational study. The study was approved by the Ethics Review Board of the University of Duisburg-Essen (approval no. 10-4521) and the samples were collected in accordance with the relevant guidelines and regulations. All patients provided written informed consent. Patient characteristics are given in Table 1.

All patients were heparinized intravenously with 400 IU/kg body weight before commencement of CPB. Venous blood samples were collected in EDTA tubes (S-Monovette, Sarstedt, Nümbrecht, Germany) at three different time points after heparinisation: before cannulation for CPB (baseline) and after aortic cannula removal following CPB. Following heparin reversal and admission to the intensive care unit after surgery a third plasma sample was collected 24 hours after surgery. Myocardial tissue samples from right atrial appendages were collected at corresponding time points before and after CPB. The biopsies were placed into cryo tubes, immediately frozen in liquid nitrogen, and stored at −80 °C until further use. Plasma was obtained by 10 minutes of centrifugation at 2000 × g and was frozen at −80 °C until miRNA isolation.

### Isolation of miRNA from plasma samples

Plasma miRNA was isolated from 400 µl plasma using the miRVana PARIS Kit (Ambion, Carlsbad, USA) following the total RNA isolation procedure according to the manufacturer’s instructions. Due to the lack of validated reference miRNAs for normalization, 25 fmol exogenous cel-miR-54 from *C. elegans* (Qiagen, Hilden, Germany) was spiked into samples immediately before miRNA isolation, as described previously^42^. This allows to control for differences in sample preparation efficiencies. Since these efficiencies are assumed to be almost identical across all samples, this spike-in control can be used additionally to detect inhibition of the miRNA quantification from any remaining heparin during sample preparations. Total RNA was eluted in 100 µl of RNase-free water and stored at −80 °C until further use.

### Isolation of miRNA from right atrial appendages

Total RNA including miRNA from right atrial samples was isolated using the RNeasy Mini Kit (Qiagen, Hilden, Germany) according to the manufacturer’s recommendations in the RNeasy fibrous tissue handbook but with slight modifications to allow the simultaneous isolation of miRNA. The tissue lysate was mixed with an 1.5-fold volume of ethanol to enhance the binding of small RNAs to the spin-column and RWT instead of RW1 wash buffer was used. The RNA was eluted in 30 µl of RNase-free water and stored at −80 °C until further use.

### Heparinase treatment of plasma miRNA samples

Heparinase I from *Flavobacterium heparinum* was purchased from Sigma-Aldrich (Munich, Germany). A stock solution of 1 U/µl was prepared in 20 mM Tris, 600 mM NaCl, 150 mM CaCl_2_, pH 7.0, and aliquots were stored at −20 °C. For heparinase I treatment 25 µl of the isolated plasma miRNA was incubated with 1 U of heparinase I in 1 × RT Buffer and 15 U RNase Inhibitor included in the Taqman MicroRNA Reverse Transcription Kit (Applied Biosystems, Carlsbad, USA) in a total volume of 30 µl at 25 °C for the indicated time.

### miRNA reverse transcription (RT) and quantitative real-time PCR (qPCR)

Reverse transcription was performed using the Taqman MicroRNA Reverse Transcription Kit and microRNA specific primer included in the Taqman MicroRNA Assays (both Applied Biosystems, Carlsbad, USA) according to the manufacturer’s instructions. Six µl from the heparinase I treatment were used as template RNA. The resulting cDNA was stored at −20 °C until further use.

qPCR was performed on an Applied Biosystems Step One Plus Real-Time PCR System using Taqman MicroRNA Assays for cel-miR-54, miR-1, miR-133a, miR-499, miR-423-5p and miR-16 and the Taqman Universal Master Mix II no UNG (all Applied Biosystems, Carlsbad, USA) in a final volume of 20 µl including 1 µl cDNA from the RT reaction as template. All samples were run in duplicate. Each run consisted of 10 min denaturation at 95 °C followed by 40 cycles of 15 sec at 95 °C and 1 min at 60 °C. Relative expression was calculated using the comparative C_T_ method according to Schmittgen and Livak^43^. The C_T_ of samples that were undetectable was set to 40 for use in expression level calculations.

### Statistical analysis

Data are presented as means ± SEM unless indicated otherwise. Data analysis was performed using SPSS V22.0 (SPSS Inc., Chicago, IL, USA) or GraphPad Prism 6 (Graph Pad Software Inc., San Diego, USA) and the level of significance was set at *p* < 0.05. To test for heparinase effects on the spike-in cel-miR-54 expression, data were analysed by either one-way analysis of variance (ANOVA) or by repeated measure ANOVA with ‘time’ as within-subject factor and ‘treatment’ as between-subject factor. To analyse endogenous miRNA expression in patient samples, data were analysed using either the Wilcoxon matched pairs signed rank test (two groups) or the Friedman test (three groups).

### Data availability

The datasets generated and analysed during the current study are available from the corresponding author on reasonable request.

## Electronic supplementary material

Supplementary Dataset 1
