# Supplementary material for: Establishment of an easy and straight forward heparinase protocol to analyse circulating and myocardial tissue micro-RNA during coronary artery-bypass-graft surgery
Source: Sci Rep. 2018 Jan 22;8:1361. doi: 10.1038/s41598-018-19748-6 (PMC5778083; doi:10.1038/s41598-018-19748-6)
Supplement: Supplementary file 1 — Supplementary Dataset 1 [file 41598_2018_19748_MOESM1_ESM.doc]

**Establishment of an easy and straight forward heparinase protocol to analyse circulating and myocardial tissue micro-RNA during coronary artery-bypass-graft surgery**

Andrea Engler^1,*^, Florian Dreja^1^, Sarah Köberle^1^, Matthias Thielmann^2^, Jürgen Peters^1^, Ulrich H. Frey^1^

^1^Klinik für Anästhesiologie und Intensivmedizin, Universität Duisburg-Essen and Universitätsklinikum Essen, Essen, 45147, Germany

^2^Klinik für Thorax- und kardiovaskuläre Chirurgie, Universität Duisburg-Essen and Universitätsklinikum Essen, Essen, 45147, Germany

*Andrea.Engler@uk-essen.de

Corresponding author:

Andrea Engler

Klinik für Anästhesiologie und Intensivmedizin

Universitätsklinikum Essen

Hufelandstr. 55

D-45147 Essen, Germany

Phone: +49 201 723 84049

Fax: +49 201 723 5949

Email: [Andrea.Engler@uk-essen.de](mailto:Andrea.Engler@uk-essen.de)

**Supplementary Table S1. Raw C_T_ values for cel-miR-54**

| **Sample No.** | **C_T_ cel-miR-54**  **without heparinase treatment** | | | **C_T_ cel-miR-54**  **with heparinase I treatment** | | |
| --- | --- | --- | --- | --- | --- | --- |
|  | **pre** | **post** | **24h** | **pre** | **post** | **24h** |
| **1** | 40 | 38.09 | 17.38 | 16.92 | 16.99 | 16.66 |
| **2** | 40 | 31.22 | 17.04 | 16.51 | 16.66 | 16.55 |
| **3** | 40 | 18.62 | 16.86 | 17.68 | 16.78 | 16.80 |
| **4** | 40 | 24.83 | 16.44 | 16.41 | 16.25 | 16.17 |
| **5** | 40 | 19.95 | 16.33 | 16.79 | 16.41 | 15.83 |
| **6** | 35.06 | 39.75 | 16.86 | 17.67 | 17.81 | 17.08 |
| **7** | 38.09 | 30.26 | 17.92 | 17.94 | 17.08 | 17.35 |
| **8** | 36.06 | 25.94 | 16.70 | 16.46 | 16.47 | 16.00 |
| **9** | 31.34 | 21.43 | 17.13 | 16.33 | 16.56 | 16.42 |
| **10** | 28.80 | 18.95 | 17.37 | 16.48 | 16.88 | 16.58 |
| **11** | 36.00 | 18.52 | 17.50 | 16.51 | 16.64 | 16.91 |
| **12** | 40 | 19.93 | 18.18 | 17.47 | 17.19 | 17.47 |
| **Mean ± SEM** | **37.1 ± 1.1** | **25.6 ± 2.2** | **17.1 ± 0.2** | **16.9 ± 0.2** | **16.8 ± 0.1** | **16.7 ± 0.1** |

|  |  |  |
| --- | --- | --- |

In samples without detectable cel-miR-54 expression the C_T_ value was set to 40 to be used in calculations.


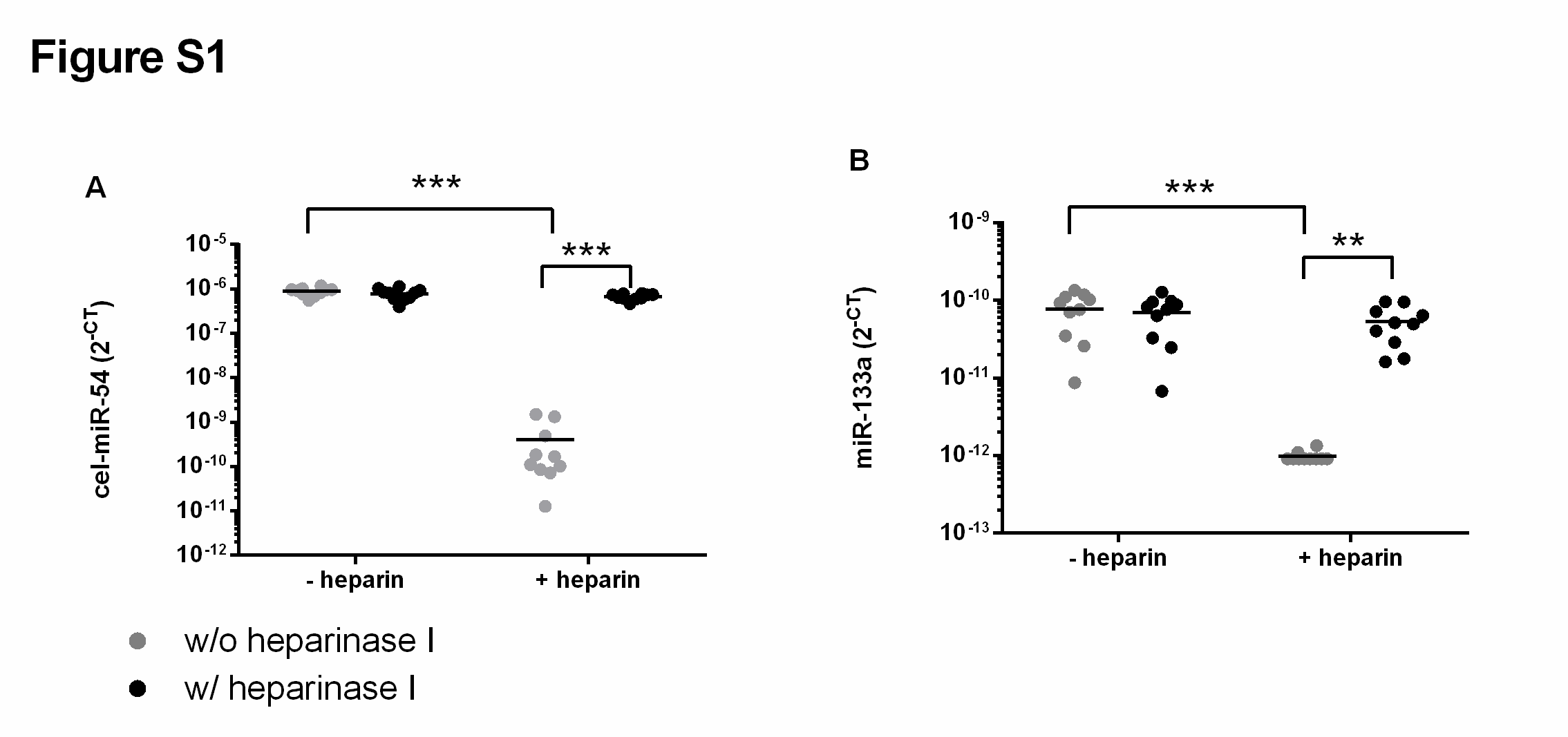


**Figure S1: Effect of heparinase I treatment on the expression analysis of different miRNAs in samples with and without heparin.** Expression analysis for cel-miR-54 (A) and miR-133a (B) was performed by qPCR (n=10). Blood samples from healthy volunteers were either spiked with heparin (+) or PBS (-) prior to plasma collection for miRNA isolation. Spiked heparin equaled the amount of heparin present in patients with extracorporeal circulation during surgery (400 IU/kg body weight). The isolated RNA from each group (+ and – heparin) was either subjected to our heparinase I treatment or was left untreated. Two way ANOVA followed by *post hoc* t-tests; **** P≤* 0.001, *** P ≤* 0.01.

**
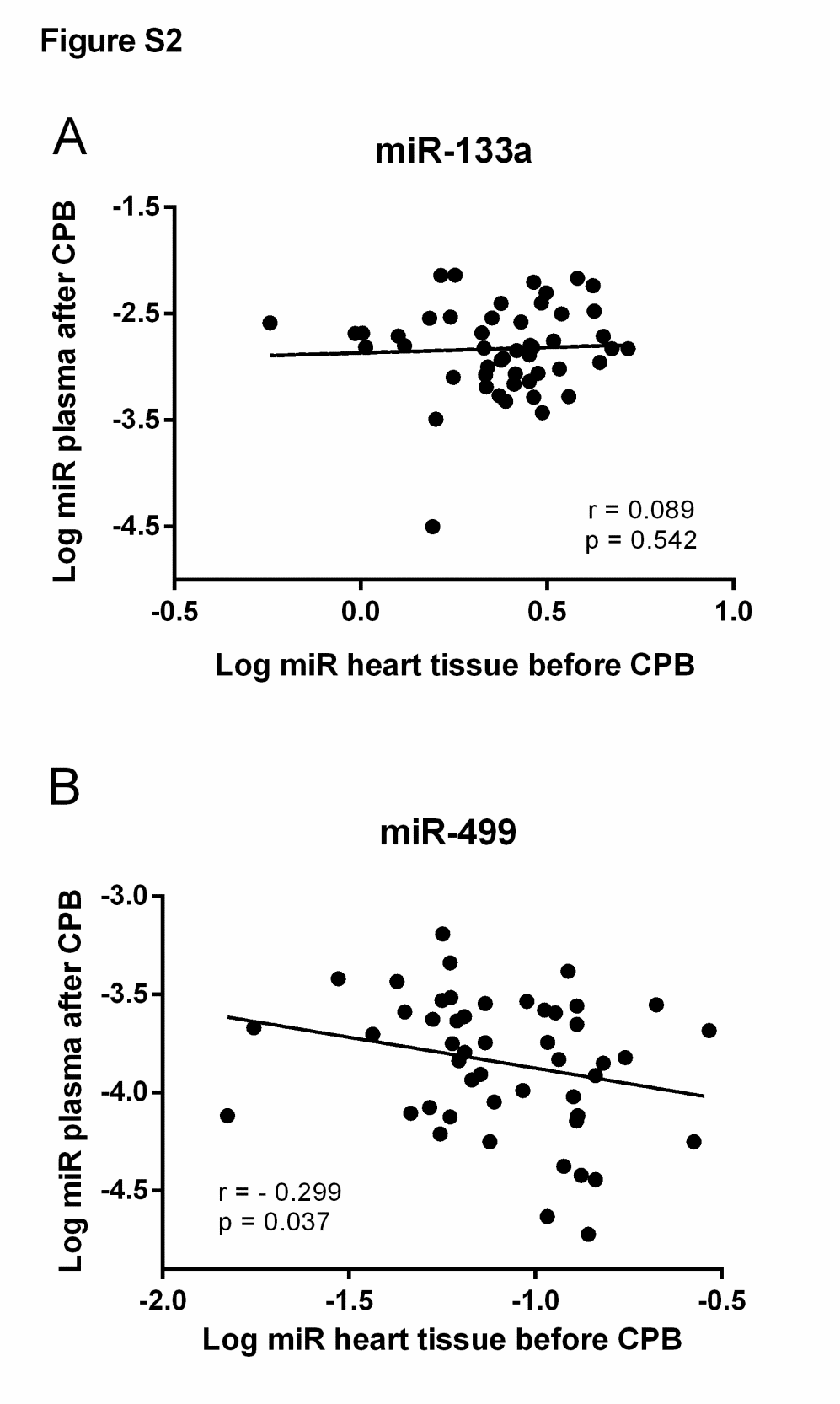
**

**Figure S2. Correlation of miR-133a and miR-499 in cardiac tissue before CPB and in plasma after CPB.** Spearman rank correlations between myocardial tissue miR-133a (A) and miR-499 (B) before CPB and their respective plasma concentrations after CPB.
